# Systematic Review and Meta-Analysis of Remineralizing Agents: Outcomes on White Spot Lesions

**DOI:** 10.3390/bioengineering12010093

**Published:** 2025-01-20

**Authors:** Ana Josefina Monjarás-Ávila, Louis Hardan, Carlos Enrique Cuevas-Suárez, Norma Verónica Zavala Alonso, Miguel Ángel Fernández-Barrera, Carol Moussa, Jamal Jabr, Rim Bourgi, Youssef Haikel

**Affiliations:** 1 Dental Materials Laboratory, Academic Area of Dentistry, Autonomous University of Hidalgo State, San Agustín Tlaxiaca 42160, Mexico; ana_monjaras@uaeh.edu.mx (A.J.M.-Á.); miguel_fernandez10334@uaeh.edu.mx (M.Á.F.-B.); 2 Department of Restorative Dentistry, School of Dentistry, Saint-Joseph University, Beirut 1107 2180, Lebanon; louis.hardan@usj.edu.lb (L.H.); rim.bourgi@net.usj.edu.lb (R.B.); 3 Facultad de Estomatología, Universidad Autónoma de San Luis Potosí, San Luis Potosí 78290, Mexico; nveroza@fest.uaslp.mx; 4 Faculty of Dentistry, University of Tours, 37032 Tours, France; carol.moussa@univ-tours.fr; 5 Division of Education, Ethics, Health, Faculty of Medicine, University of Tours, 37044 Tours, France; 6 Private Practice (Healthcare Center), L-1740 Hollerich, Luxembourg; ortholux.jabr@gmail.com; 7 Department of Biomaterials and Bioengineering, INSERM UMR_S 1121, University of Strasbourg, 67000 Strasbourg, France; 8 Department of Endodontics and Conservative Dentistry, Faculty of Dental Medicine, University of Strasbourg, 67000 Strasbourg, France; 9 Pôle de Médecine et Chirurgie Bucco-Dentaire, Hôpital Civil, Hôpitaux Universitaire de Strasbourg, 67000 Strasbourg, France

**Keywords:** casein phosphopeptide–amorphous calcium phosphate, hardness, lesion depth, meta-analysis, remineralization, varnish, white spot lesion

## Abstract

Dental caries is a widespread issue impacting global oral health. White spot lesions, the earliest stage of caries, compromise enamel’s esthetics and integrity. Remineralization therapies, both fluoride and non-fluoride based, aim to restore enamel, but limited comparative data exist on their effects on lesion depth and microhardness. Thus, the aim of this systematic review was to evaluate the efficacy of remineralizing agents on lesion depth and microhardness of human teeth. The literature search included the following five databases: PubMed, Web of Science, Scielo, SCOPUS, and EMBASE from the period 2012 to October 2022. Studies evaluating lesion depth and microhardness in human teeth after the application of a remineralizing agent were considered for review. The meta-analysis was performed using RevMan 5.4 (The Cochrane Collaboration, Copenhagen, Denmark). A random effect model was used to pool estimate of effect and its 95% confidence intervals (CIs) for surface microhardness and depth lesion. Subgroup analyses were performed considering the presence of fluoride or not in the remineralization agent. Thirty-three studies were included in the qualitative review. Of these, twenty-six studies were included in the meta-analysis. The main risks of bias associated with the studies included a lack of blinding of the test operator and failure to obtain sample size. To conclude, fluorinated agents are more effective in remineralizing artificially induced white spot lesion than non-fluoride remineralizing agents.

## 1. Introduction

A white spot lesion (WSL) is the initial clinical manifestation of an early caries lesion in dental enamel, presenting with subsurface porosity. It is characterized by its opacity, reduced fluorescence brightness, and loss of enamel translucency [1]. The white appearance of these lesions is due to an optical phenomenon caused by the loss of minerals on the surface and below the surface, modifying the result in the refractive index [2].

When a WSL progresses into a cavitated lesion, various strategies are considered to repair or reverse the earliest signs of enamel demineralization. According to the International Caries Detection and Assessment System (ICDAS), WSLs are classified as Code 1 when visible only on dry surfaces and Code 2 when visible on wet surfaces. Remineralization therapy has emerged as a popular approach for treating these lesions. Notably, under physiological conditions, factors in the oral environment—such as saliva—play a protective role in maintaining a balance in the demineralization–remineralization (DES-RE) process on enamel surfaces, which is influenced by fluctuations in pH levels [3].

A WSL is considered highly responsive to early intervention aimed at reversing demineralization. Treatment goals focus on restoring the lesion’s depth, hardness, and mineral content, which are impacted by this initial stage of enamel injury. The mineral-deficient substrate of a WSL makes it susceptible to therapies designed to halt progression and encourage remineralization, potentially returning the enamel to a healthier, more resilient state [4]. The application of fluoride is one of the strategies to prevent and control the appearance of carious lesions, since it interacts with saliva on the surface and adjacent subsurface layers of tooth enamel, which combines with phosphate and calcium ions, to form fluorhydroxyapatite crystals, optimizing remineralization. Many reports indicate that fluoride is the gold standard for preventing and controlling WSL [3,5]; however, various remineralization therapies have been developed to address white spot lesions (WSLs) using advanced agents like casein phosphopeptide–amorphous calcium phosphate (CPP-ACP), arginine, xylitol, Novamin technology, and nano-hydroxyapatite. These agents, either independently or in combination with fluoride, show promise by acting as reservoirs of calcium and phosphate. This reservoir effect supports enamel remineralization by delivering essential ions directly to the lesion, thereby promoting the restoration of mineral content and potentially reversing the effects of early demineralization [6,7]. As the number of products to choose from increases and with limited public information about the preventive benefits of fluoride on dental health, people may turn to toothpastes with low remineralizing capacity and unproven benefits, often due to concerns over fluoride cytotoxicity.

There is a meta-analysis study that explores the differences between remineralizing agents from clinical studies [7], while leaving aside the large number of in vitro contributions that have been reported. Therefore, the objective of this study was to compare the effectiveness of remineralizing agents with fluoride and without fluoride on artificial caries lesions. This aims to update and reinforce the information available regarding therapeutic options for white spot remineralization and how these agents affect the composition and physical-mechanical properties of enamel. This comparison will help discern between fluoride and non-fluoride agents and contribute to establishing clinical protocols for the remineralization of WSLs. Accordingly, the null hypothesis tests that there is no significant difference in the effectiveness of remineralizing agents with fluoride compared to those without fluoride on artificial caries lesions.

## 2. Materials and Methods

Preferred Reporting Items for Systematic Reviews and Meta-Analyses statement guidelines were followed for review [8]. Registration was carried out at the Open Science Framework platform under the identifier 10.17605/OSF.IO/HR2VG. The Population, Intervention, Comparison, Outcome, and Study design method are presented in Table 1.

### 2.1. Search Strategy and Selection of Studies

A comprehensive electronic search for in vitro studies was conducted in five databases, and the search results are summarized in Table 2. Studies evaluating WSL depth and micro-hardness in human teeth from 2012 to October 2022 were considered for review. The inclusion criteria encompassed articles assessing the effect of different remineralization agents using methods such as Polarized Light Microscopy (PLM), Confocal Microscopy, and Vickers Hardness testing. Case reports, clinical studies, abstracts, editorials, review and meta-analysis articles, articles not in English, studies using animal tooth samples, and those without a control group were excluded. It is important to note that studies employing Energy-Dispersive X-ray Spectroscopy (EDX) were included only in the systematic review to aid in the characterization of remineralizing agents but were excluded from the meta-analysis due to their inability to assess lesion depth and microhardness. Reference articles were retrieved and exported to Mendeley Desktop 1.13.3 software (Elsevier. Mendeley Ltd., London, UK) [9].

### 2.2. Data Extraction

Two reviewers (A.J.M.-Á and R.B.) screened the titles and abstracts of each study based on the criteria and extracted data. They independently re-verified the full text of the selected studies. Any disagreement between the two was resolved by a third reviewer (C.E.C.-S.). Data collected for each study included information related to the year of publication, authorship, remineralizing agent used, control group, DES-RE protocol, artificial caries formation, pH cycling process, and type of test applied.

### 2.3. Risk of Bias Assessment

A customized risk assessment tool was designed using the Office of Health Assessment and Translation risk of bias tool [10] and checklist for reporting in vitro study guidelines [11]. All included studies were independently assessed by two review authors who verified the identification specifications of the articles. Each item was classified as low risk, unclear, and high risk. The Cochrane risk of bias assessment tool was used to generate the graph and summary [12].

### 2.4. Statistical Analysis

Data were analyzed using RevMan 5.4 (The Cochrane Collaboration, Copenhagen, Denmark). Heterogeneity between estimates was assessed using the Cochrane test (I^2^ test) at α = 0.10. I^2^ > 50% indicated high heterogeneity [13]. A *p* (two-tailed) < 0.05 was set to test the hypothesis. The effectiveness of remineralizing agents with fluoride versus without fluoride on the decrease in lesion depth and change in surface microhardness of artificial WSL of primary and permanent teeth over the period of 5 to 21 days was evaluated. The treatment effect was summarized by means difference and standard deviations. A randomized effects model was used to combine studies due to heterogeneity across studies. A pooled estimate of the effect and its 95% confidence intervals (CI) were calculated for lesion depth and surface microhardness. Data from eligible studies were extracted into RevMan software and a forest plot was generated for graphical presentation. Meta-analysis was not performed for the mineral loss/gain, roughness, and color parameters due to differences in measurement results.

## 3. Results

### 3.1. Study Selection and Description

**Figure 1:**
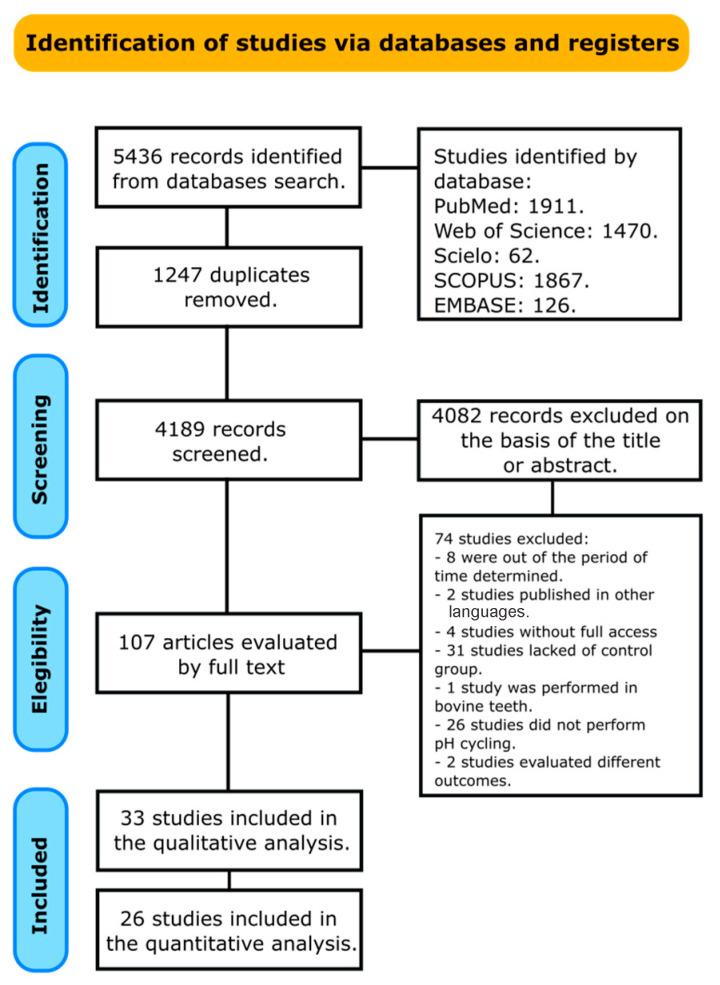
Diagram of review methodology.

Through the literature search, 5436 studies were identified, including 1247 duplicates. In total, 4189 articles were identified after excluding duplications. A total of 4082 articles evaluating the efficacy of remineralizing agents were excluded after reading abstract. Full-text articles were retrieved for 107 relevant studies. From these, 74 articles were excluded after full-text reading. Finally, 33 studies that met the inclusion criteria were considered (Figure 1). The review evaluated two different outcomes: lesion depth and surface microhardness. When evaluating the effectiveness of remineralizing agents in reversing WSL, studies often assess the outcomes over a period of 5 to 21 days in comparison to control groups. This time frame allows researchers to monitor the changes in enamel remineralization and texture improvement. Results of 26 studies were synthesized using forest plot, and 7 studies were analyzed only qualitatively.

### 3.2. Study Characteristics

Thirty-three relevant studies published between 2012 and 2022 (October) were found. Ten studies were reported from India, nine from Thailand, three from China, three from Turkey, and one from Poland, Serbia, Alappuzha, Iran, Brazil, Germany, USA, and Pujab. The studies were conducted on 1057 extracted permanent human teeth, 458 deciduous teeth, and 138 that only mentioned extracted humans. These studies reported intervention on molars, premolars, and anterior teeth. The methods for the evaluation of surface microhardness mostly included Knoop and Vickers Hardness. To evaluate the depth of the lesion, a polarized light microscope and Diagnodent were used. In studies evaluating the effectiveness of remineralizing agents for WSL, the test group typically consists of any remineralizing agent applied to the lesions. These are compared to one or more control groups, which may include options such as no treatment, or the use of distilled and deionized water. The follow-up period ranged from 5 to 21 days (Table 3 and Table 4).

### 3.3. Risk of Bias Assessment

**Figure 2:**
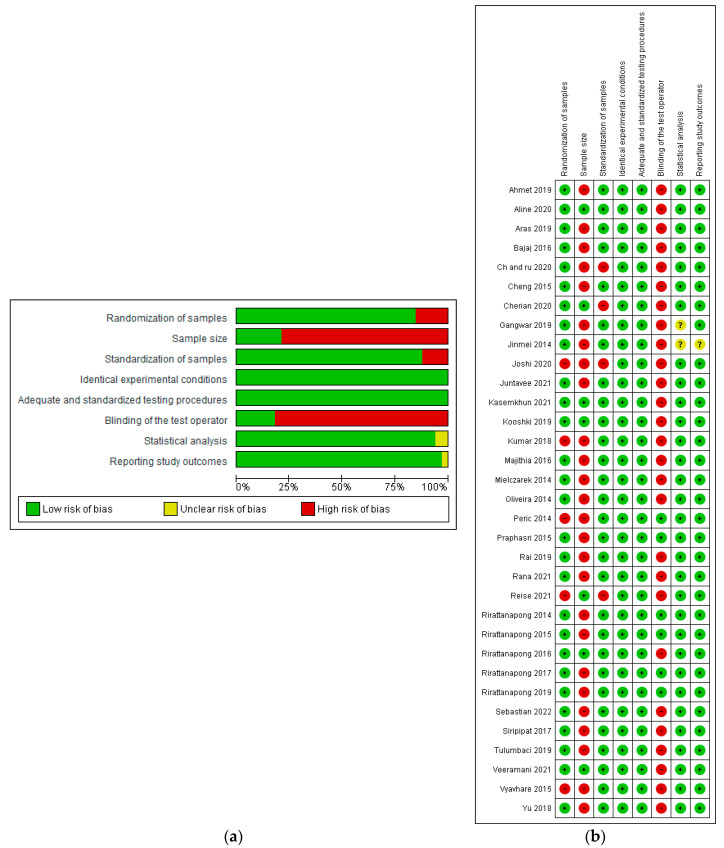
Cochrane risk of bias of the included studies (**a**) graph, (**b**) summary [14,15,16,17,18,19,20,21,22,23,24,25,26,27,28,29,30,31,32,33,34,35,36,37,38,39,40,41,42,43,44,45,46].

A score of 1 was assigned to each risk of bias item, if mentioned. The overall risk level of each study was subsequently classified as low risk, moderate risk/unclear risk, and high risk (if the score was 6 or more, 3 or more, and <3 of the eight categories, respectively). Scores were averaged for each included study. No score was given when the risk of bias element was not clearly mentioned. For example: there is no mention of sample size calculation; blinding and multiple samples prepared from the same sample [16]. Overall, 26 [17,18,19,20,21,22,23,24,25,26,27,28,29,30,31,32,33,34,35,36,37,38,39,40,41,42,43] of the 33 included studies [44,45,46,47,48,49,50] were at moderate risk. The types of bias of the included studies are summarized in Figure 2a,b. The main risks of bias associated with these studies included not mentioning aspects such as sample randomization, sample size calculation, sample standardization, and operator blinding.

### 3.4. Summary of Results—Effect of the Interventions

#### 3.4.1. Lesion Depth

##### Primary Teeth Treated with Fluoride Agents

**Figure 3:**
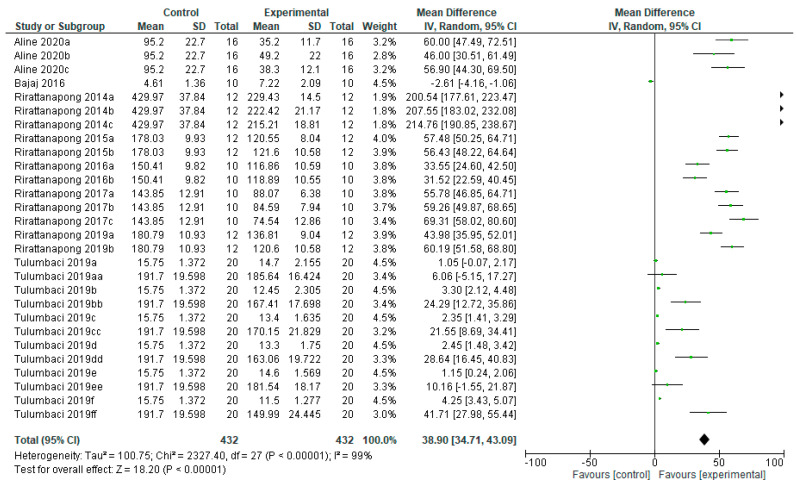
Forest plot of comparison: Lesion Depth Primary Teeth, Fluoride Agents [14,17,35,36,37,38,39,40,43].

There is a statistically significant difference (*p* < 0.00001) in the effectiveness of remineralizing agents with fluoride on primary teeth, compared to the control group (Figure 3). Of the eight studies, one study [17] did not show results in favor of the remineralizing agent, and one [43] did not show any difference between groups. Fluorinated agents showed a superior remineralizing effect, reducing the lesion depth compared to no treatment [14,35,37,38,39,40,43].

##### Primary Teeth Treated with Fluoride-Free Agents

**Figure 4:**
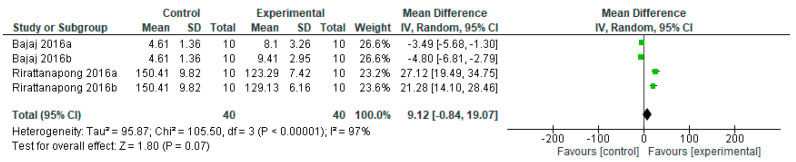
Forest plot of comparison: Lesion Depth Primary Teeth, Fluoride-Free Agents [17,38].

Fluoride-free remineralizing agents behave the same as the control group on primary teeth, without a statistically significant difference (*p* = 0.07) (Figure 4). Of the two studies analyzed, one study [17] shows results in favor of the control group, and the results of one study [38] favor the experimental group.

##### Permanent Teeth Treated with Fluoride Agents

**Figure 5:**
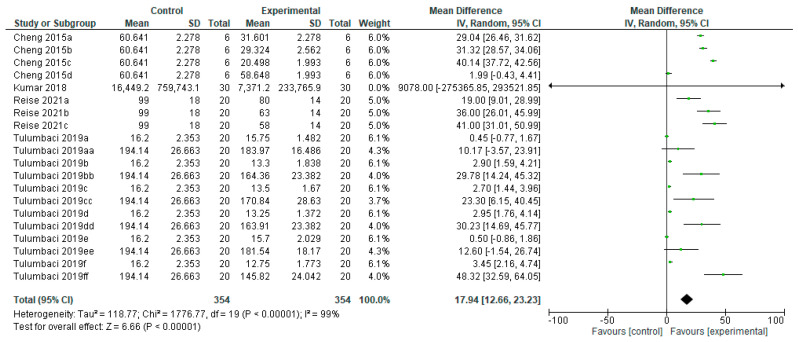
Forest plot of comparison: Lesion Depth Permanent Teeth, Fluoride Agents [19,26,34,43].

The remineralization of the lesion depth of the WSL is favored by agents with fluoride in permanent teeth, when compared with the control group, with a statistically significant difference (*p* = 0.00001) (Figure 5). The results of four studies were compared. Three articles [19,26,43] are favored in the same way by the control and experimental groups. The results of different agents from three studies [19,34,43] favor the experimental group.

##### Permanent Teeth Treated with Fluoride-Free Agents

**Figure 6:**
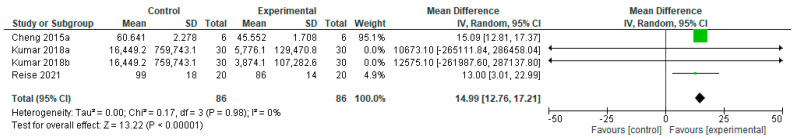
Forest plot of comparison: 3 Lesion Depth Permanent Teeth, outcome: 3.2 Fluoride-Free Agents [19,26,34].

Fluoride-free agents show superior efficacy on the depth of injury in permanent teeth, when compared with the control group (Figure 6), showing a statistically significant difference (*p* = 0.00001). The results of two studies [19,34] favor the experimental group, while the results of a study [26] do not show difference between groups.

#### 3.4.2. Surface Microhardness

##### Primary Teeth Treated with Fluoride Agents

**Figure 7:**
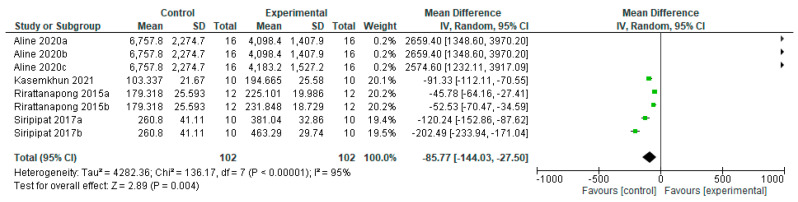
Forest plot of comparison: Microhardness Primary Teeth Fluoride Agents, Fluoride Agents [14,24,37,42].

The microhardness of primary teeth treated with remineralizing agents containing fluoride does not show significant improvement when compared to control groups (*p* = 0.10, Figure 7). The results of three studies demonstrated positive outcomes with the use of remineralizing agents [24,37,42], while one study found no significant difference between the groups treated with these agents and the control group [14].

##### Primary Teeth Treated with Fluoride-Free Agents

**Figure 8:**
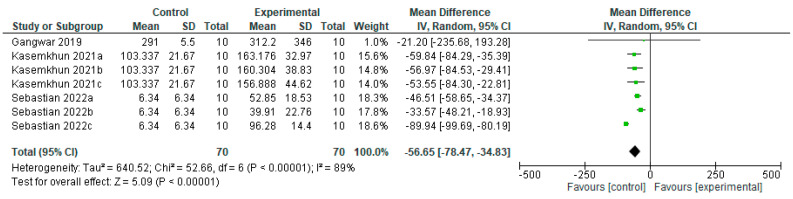
Forest plot of comparison: Microhardness Primary Teeth Fluoride Agents, Fluoride-Free Agents [21,24,41].

Primary teeth treated with fluoride-free agents do not benefit in changes in microhardness when compared to the control group (Figure 8), showing a statistically significant difference (*p* = 0.00001). Six results of different agents in two studies [24,41] favor the control group, and one study [21] does not favor any group.

##### Permanent Teeth Treated with Fluoride Agents

**Figure 9:**
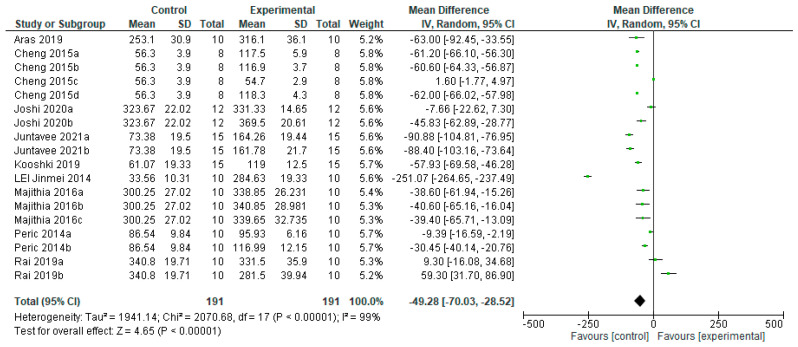
Forest plot of comparison: Microhardness Permanent Teeth Fluoride Agents, Fluoride Agents [16,19,22,23,25,27,28,31,32].

The efficacy of remineralizing agents with fluoride to permanent teeth on WSL microhardness favors the control group when compared to the experimental group (Figure 9), with a statistically significant difference (*p* = 0.00001). From the pooled data of nine studies, in three [19,22,31] no difference was obtained between groups, one study [32] favors the experimental group, and eight studies [16,19,22,23,25,27,28,31] benefit more from the control group.

##### Permanent Teeth Treated with Fluoride-Free Agents

**Figure 10:**
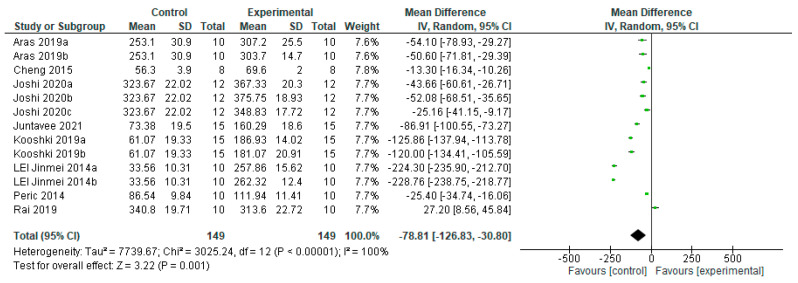
Forest plot of comparison: Microhardness Permanent Teeth Fluoride Agents, Fluoride-Free Agents [16,19,22,23,25,27,31,32].

The microhardness of the WSL in permanent teeth treated with fluoride-free agents benefited from the control group compared to the experimental group (Figure 10), with a significant statistical difference (*p* = 0.001). Data from eight studies were included for this analysis, of which one [32] is favored by the experimental group and seven [16,19,22,23,25,27,31] are favored by the control group.

## 4. Discussion

This systematic review with meta-analysis highlighted the important remineralizing effect of fluorinated agents compared to non-fluorinated agents in primary and permanent teeth. The term remineralization has been used to describe mineral gain, including mineral precipitation on glazed surfaces [47,51]. Most in vitro studies have shown that fluoride-based remineralizing agents are more effective at reversing WSL than non-fluorinated agents. However, a few studies have presented contradictory findings regarding the effectiveness of these agents.

The results of this study demonstrate the effectiveness of remineralizing agents on the depth of WSL in permanent teeth, both with fluorinated and non-fluorinated agents. However, primary teeth showed no significant effect on lesion depth when non-fluoride agents were applied. Thus, the null hypothesis tested in this manuscript is partially rejected. It is important to note that there are fewer studies examining the effectiveness of remineralization with non-fluorinated agents.

Greater effectiveness has been demonstrated with the combination of remineralizing agents, since it increases the incorporation of fluoride into saliva, plaque, and superficial enamel, unlike agents that only contain fluoride [48].

Studies of primary teeth included in this review involve applications of sodium fluoride in toothpaste, varnish, and mouthwash, as well as the combination of agents such as fluoride with functionalized Tri-calcium Phosphate (fTCP) in toothpaste, varnish, mouthwash, Fluoride with Casein Phosphopeptide Amorphous Calcium Phosphate, and varnish, as well as fluoride with novamin technology, which is an active biocrystal containing Calcium Sodium Phosphosilicate (CSP) and stannous fluoride. On the other hand, the agents used for permanent teeth mostly come in fluoride-based toothpaste, CPP-ACP, CSP, and other components such as arginine.

Fluoride-based agents are largely studied, and a variety of combinations exist, causing substantial clinical heterogeneity. Tooth hardness depends on the ratio between demineralization and remineralization [49]. According to the results of microhardness, the meta-analysis showed significant differences in the results that favor the control group, which demonstrates that agents with fluoride and without fluoride, in paste, varnish, or rinse, do not show changes in the lesion of the WSL in a period of 5 to 21 days, whether on primary or permanent teeth. It is also noted that more studies have been carried out on the effectiveness of remineralizing agents with fluoride and without fluoride on the microhardness of the WSL of permanent teeth unlike primary teeth.

Shanbhog et al., demonstrated that a 1400 ppm fluorinated toothpaste is more effective than a 1000 ppm fluorinated toothpaste and non-fluorinated toothpaste in increasing the surface microhardness of enamel [50]; unlike the results of this review, fluoride-based toothpastes failed to effectively remineralize and recover the microhardness of the WSL in primary and permanent teeth.

The limitations of this study stem from the fact that the pieces where the samples were obtained are from recently extracted central, premolar, and permanent teeth. It was verified that the primary caries lesion will be caused artificially mostly under the same protocol, according to the Carbopol method as described by White [51], where the same solution of calcium chloride and acetic acid adjusted to the same level of pH for either 72 h or 96 h, at 37 degrees Celsius. However, some variations in methodology included the use of lactic acid, hydrochloric acid, and phosphoric acid, which led to minor discrepancies in the initial caries lesion formation. These lesions are referred to in some studies as non-cavitated superficial caries or WSL. It is worth mentioning that the in vitro preparation of a primary caries lesion in the enamel provides a lesion of approximately the same depth in all samples, which provides a standard to compare between different remineralizing agents [52]. It was confirmed that all studies used the pH-cycling model as a method to simulate plaque acid challenges in the demineralization process. This approach provides a valid simulation of caries progression and is considered more reliable for replicating the clinical conditions of the DES-REM process [4]. Despite the use of different numbers and durations of cycles in some studies, all samples were stored in artificial saliva to replicate the remineralizing conditions of the oral environment and were rinsed with deionized water between each cycle change to improve standardization. Another limitation stems from the units used to present results for the measured variables; while some were reported in nanometers, others used microns. Additionally, various methods such as the Knoop and Vickers Hardness tests for microhardness, and LPS or Diagnodent for lesion depth, contributed to methodological diversity. The variations in lesion locations, types of agents, and treatment frequencies introduced further heterogeneity when analyzing results. A quality assessment of evidence in the meta-analysis can be found in Table 3 (characteristics of the included studies) and Figure 2 (risk of bias for each study). Rigid inclusion criteria during the selection process helped to minimize potential bias.

Currently, there is still a report that states that the fluoride content in oral hygiene agents can cause fluorosis and superior weakening of the tooth, and because of that, fluoride-free therapeutic options have been provided [53]; therefore, authors have made efforts to compare the remineralizing efficacy of these new agents and, thus, avoid the risk of fluoride cytotoxicity.

It has been shown that agents in paste, gel, varnish, or mouthwash forms face greater difficulty in acting effectively on pit and fissure lesions than on smooth surface lesions due to the complexity of the anatomical structure [54]. However, in clinical practice, pits and fissures can retain these agents for extended periods regardless of their form, which enhances the remineralizing effect and offers greater therapeutic benefit [47].

On the other hand, under oral conditions, Casein Phosphopeptide can bind to the biofilm and act as a pool of slow-release calcium and phosphate ions; being a factor that probably led to lower values of CPP-ACP remineralization, since in an in vitro study this complex oral environment is difficult to replace [55]. Despite this, the remineralizing properties of CPP-ACP have been exhibited in a prior research study [56].

Regarding agents with nano-hydroxyapatite, which is one of the most biocompatible bioactive materials due to the presence of its nanometric-sized particles that are similar to tooth enamel in morphology and crystalline structure [57], it has been reported to remineralize artificial caries lesions after its incorporation into toothpaste and mouthwash. The results of this study show that agents containing hydroxyapatite and nano-hydroxyapatite do not favor the remineralization of the lesion depth in primary teeth, nor microhardness in primary or permanent teeth in vitro. Of all the authors included in the meta-analysis, only Rai in 2019 [32] obtained greater efficacy with the experimental group than with the control group, on the microhardness of the initial caries lesion in permanent teeth (HA340.8 ± 19.71 Control313.6 ± 22.72) (Figure 10).

As in another study [58], the results of the meta-analysis show that CPP-ACP + fluoride has the greatest remineralizing power, followed by TCP + Fluoride. Therefore, agents containing fluoride, either alone or in combination, produce greater remineralization of the early caries lesion, over its depth and not over its hardness, in primary and permanent teeth, than the agents containing calcium phosphate, hydroxyapatite, arginine, xylitol, or some other agent with remineralizing action alone.

Resin infiltrants have shown promise in restoring WSLs by arresting caries progression and enhancing enamel esthetics. Incorporating bioactive materials, such as hydroxyapatite, calcium phosphate-based compounds, and bioactive glass, into resin infiltrants offers additional benefits. These materials can promote remineralization by releasing calcium and phosphate ions, which facilitate the repair of demineralized enamel [59]. Hydroxyapatite, in particular, mimics the mineral composition of enamel and dentin, aiding in the restoration of structural integrity [60]. Similarly, bioactive glass has demonstrated the ability to form a hydroxycarbonate apatite layer upon exposure to saliva, enhancing enamel repair and resistance to future demineralization [61]. The integration of these materials into resin infiltrants represents an innovative approach for both esthetic restoration and long-term preservation of enamel integrity in WSL management.

While fluoride remains a cornerstone in enhancing enamel microhardness through remineralization, other materials have also demonstrated significant potential. CPP-ACP enhances remineralization by stabilizing calcium and phosphate ions, forming a protective layer on the enamel surface [62]. Similarly, bioactive glass, when exposed to saliva, releases ions that form hydroxycarbonate apatite, effectively repairing demineralized enamel [63]. Nano-hydroxyapatite particles, due to their similarity to natural enamel, have shown the ability to integrate into enamel microstructure and improve surface hardness [60]. Studies comparing fluoride-based products, such as Remin Pro, under pH-cycling and storage in artificial saliva conditions, indicate that these materials effectively enhance enamel microhardness in primary teeth, albeit to varying degrees depending on formulation and application protocols [64]. Incorporating these alternative materials into clinical strategies offers a complementary approach to fluoride, particularly in pediatric dentistry.

A notable limitation of this review is the inclusion of studies that utilized distilled or deionized water rather than artificial saliva, which may not fully replicate the oral environment. Artificial saliva, by mimicking the ionic composition and buffering capacity of saliva, provides a more realistic model for evaluating remineralization and demineralization processes [65]. Several studies have demonstrated that pH-cycling models, combined with the use of artificial saliva, better reflect clinical conditions. For example, Featherstone et al. [66] highlighted the effectiveness of fluoride treatments in a pH-cycling environment designed to simulate periods of acid challenge and remineralization. Similarly, Amaechi et al. [67] showed that maintaining samples in artificial saliva enhances the interpretation of remineralization efficacy by providing a continuous supply of calcium and phosphate ions. Future studies should prioritize such models to improve clinical relevance and comparability of findings.

Caries is a significant global health issue, affecting approximately 60% to 90% of the population. Recent studies now include WSLs as quantifiable indicators of caries, whereas previous reports primarily focused on clinically visible manifestations of caries in moderate to severe forms, typically only documenting cavitated lesions classified as ICDAS 5 and 6. This expansion of criteria allows for earlier detection and intervention, offering a more comprehensive understanding of caries progression and prevention strategies in dental health [68]. The WSL is preventable and reversible. This study aims to provide a viable therapeutic measure to inactivate the lesions by remineralizing them so that they do not progress to cavitation, thus, reducing the very high numbers of dental destruction, due to the sequelae of the carious lesion, in children and adults. Likewise, the dental professional must guide the population so that they can reduce risk factors and maintain their oral health in stable conditions, as well as adequate functionality and esthetics.

## 5. Conclusions

The analysis of the data on lesion depth and surface microhardness of the enamel in vitro shows that fluoride-based remineralizing agents and their combination potentiate remineralizing efficacy by reducing the depth of the initial caries lesion in primary and permanent teeth; however, remineralizing agents, in general, do not achieve significant changes regarding the microhardness that was affected by WSL on tooth enamel. Based on the results of this study, the use of fluoride-based remineralizing agents is suggested in children and adults to control the progression of the primary caries lesion that will lead to the tooth structure not cavitating.

## Figures and Tables

**Table 1:** Study design with PICOS format.

Component	Description
Population	White Spot Lesion on permanent and deciduous teeth
Intervention	Chemical Remineralizing agent (non-organic agents).
Comparison	Distilled and deionized water without treatment with subsequent application of distilled water
Outcome	Depth of injury Microhardness
Study desing	In vitro studies

**Table 2:** Search strategy of the study.

Database	Search Strategy	Articles Retrieved
PubMed	(White spot lesion on enamel OR Early enamel lesion OR Artificial enamel caries OR) AND (Enamel Remineralization) AND (Lesion depth OR Microhardness)	1911
Web of Science	TS = (White spot lesion on enamel OR Early enamel lesion OR Artificial enamel caries) TS = (Enamel Remineralization) TS = (Lesion depth OR Microhardness)	1470
Scielo	TS = (White spot lesion on enamel OR Early enamel lesion OR Artificial enamel caries) TS = (Enamel Remineralization) TS = (Lesion depth OR Microhardness)	62
SCOPUS	ALL (“White spot lesion on enamel” OR “Early enamel lesion” OR “Artificial enamel caries” AND ALL (“Enamel Remineralization”) AND ALL (“Lesion depth” OR “Microhardness”)	1867
EMBASE	‘White spot lesion on enamel’ OR ‘Early enamel lesion’ OR ‘Artificial enamel caries’ ‘Enamel Remineralizing’ ‘Depth of injury’ OR ‘Microhardness’	126

**Table 3:** Characteristics of included studies.

Author/Year/Country	Sample Teeth	Making White Spot Lesion	Intervention Group (*n*)	Comparison Group (*n*)	Follow- Up Remineralization Period	Examination Methods
Aline Laignier Soares/2020/Brazil [14]	Human primary teeth	early caries lesions	5% NaF (16) 5% NaF with CPP-ACP (16) 5% NaF with TCP (16)	purified water (16)	8 days	Polarized Light Microscopy (PLM) Knoop Hardeness (KHN)
Aras, A./2019/Turkey [15]	Fifty freshly extracted third molar teeth	artificial incipient enamel caries	CPP-ACPF MI Paste Plus cream (10) NovaMin-fluoride toothpaste (10) Xilitol–fluoride cream (10)	without treatment and washed with desionized wáter (10)	9 days	Vickers Microhardness (VHN)
Aras, A./2019/Turkey [16]	Fourty-four extracted, impacted human wisdom teeth	artificial incipient enamel caries 9 days	Fluoride gel: Topex^®^ APF gel. (10) CPP-ACP MI Paste (10) CPP-ACPF MI Paste Plus (10) Sensodyne- NovaMin-Fluoride toothpaste (10) Ksilitol-Hydroxyapatite-Fluoride cream (10) Ozone-Fluoride (10)	without treatment and washed with distilled wáter (10)	9 days	Laser Fluorescence (DIAGNOdent)
Bajaj, M./2016/India [17]	Ten primary molars	demineralizing solution for 96 h to produce artificial caries like lesions, approximately 150–200 μm thick	CPP-ACP MI Paste (10) f-TCP (Clinpro Tooth Creme) (10) Remin Pro (10)	without treatment and washed with desionized water (10)	10 days	Polarized Light Microscopy (PLM)
Chandru, T./2020/India [18]	Sixty-two permanent maxillary and mandibular incisors	initial enamel lesions, after 72 h of demineralization	Colgate Sensitive Plus^®^ Pro-Argina (15) Regenerate Enamel Science tooth paste NR-5 Technology (15) BioRepair^®^ tooth paste (15) Spectrum Reagents and Chemicals (15)	deionized wáter (15)	12th days	Vickers Hardness (VHN)
Cheng, X./2015/China [19]	Sixty human permanent molars	early artificial enamel carious lesions	0.5% arginine solution (8) Arginine/NaF solution (2.5% arginine, 500 ppmF) (8) NaF solution (500 ppmF) (8) Toothpaste A slurry (8) Toothpaste B slurr (8)	deionized water (8)	10 days	Polarized Light Microscopy (PLM) Knoop Hardness (KHN)
Cherian, N.M./2020/India [20]	Forty extracted human permanente premolar teeth	Artificial lesión for 4 days	CPP-ACP MI Paste (10) Remin Pro (10) f-TCP (Clinpro Tooth Creme) (10)	Without treatment and washed with desionized wáter (10)	21 days	Scanning electron microscopy with an energy dispersive X-ray analysis
Gangwar, A./2019/India [21]	Forty freshly extracted sound human primary anterior teeth	artificial carious lesions	Novamin (10)	without treatment and washed with desionized wáter (10)	10 days	Vickers Hardness (VHN)
Joshi, C./2020/India [22]	Sixty teeth Permanent premolars	enamel lesions for 48 h	BAG Novamin nHAp (12) f-TCP (Clinpro Tooth Creme) (12) GSE: Fluoride (1000 ppm) (12)	distilled wáter (12)	21 days	Vickers Microhardness (VHN)
Juntavee, A./2021/Thailand [23]	Sixty extracted human premolars	carious lesion on the enamel for 12 h	Nano-HA toothpaste (15) f-TCP (Clinpro Tooth Creme) (15) Fluoride toothpaste (1000 ppm) (15)	deionized wáter (15)	10 days	Polarized light microscopy (PLM)
Kasemkhun, P./2021/Thailand [24]	Fifty coronal parts of sound primary incisors	for 4 days to produce 60–100 μm depth of carious lesion	0.22% sodium fluoride (NaF) or 1000 ppm F toothpaste (10) non-fluoridated toothpaste CaGP and CL (10) CPP-ACP MI Paste (10) non-fluoridated toothpaste containing NHA (10)	deionized water (10)	7 days	Vickers Hardness (VHN)
Kooshki, F./2019/Iran [25]	Sixty intact human pre-molars	caries-like lesions on the enamels for two days	Duraphat varnish (15) Nano Paste (15) CPP-ACP MI Paste (15)	without treatment and washed with desionized wáter (15)	10 days	Vickers Hardness (VHN)
Kumar, K./2018/Alappuzha [26]	Thirty maxillary first and second premolars	lesion formation for 72 h	Monofluorophosphate dentifrice (30) CPP-ACPF MI Paste Plus (30) Calcium Sodium Phosphosilicate (CSP) (30)	without treatment and washed with distilled wáter (30)	5 days	Confocal Laser Microscopic Analysis
Lei/2014/China [27]	Fifty caries-free human upper premolars	enamel lesion formation (34% phosphoric acid for 15 s)	CPP-ACP MI Paste (10) Calcium Sodium Phosphosilicate (CSP) (10) Sodium Fluoride varnish (NaF) (10)	distilled and deionized wáter (10)	10 days	Vickers Hardness (VHN)
Majithia, U./2016/India [28]	Forty premolars	enamel lesions	Flor-Opal Varnish White (10) Premier Enamel Pro (10) MI Varnish (10)	without treatment and washed with desionized wáter (10)	5 days	Vickers Hardness (VHN)
Mielczarek A./2014/Poland [29]	Ninety human extracted teeth	enamel lesions according to the Carbopol method as described by White	NHAPF–Apa Cared toothpaste (30) F–Blend-a Mede toothpaste (30)	distilled wáter (30)	3-week	Vickers Hardness (VHN)
Oliveira, G.M. S./2014/United States [30]	Thirty-five extracted human third molars	artificial white spot lesions	CPP-ACP MI Paste (35) F5000 ControlRx (35) CPP-ACPF MI Paste Plus (35)	without treatment and washed with desionized wáter (35)	30 days	QLF análisis
Peric, T.O./2014/Serbia [31]	Ten buccal or lingual surfaces obtained from sound extracted third molars	artificial carious lesion	CPP-ACP MI Paste (10) CPP-ACPF MI Paste Plus (10) 0.05% NaF (Curasept ADS 205) (10)	without treatment and washed with distilled water (10)	10 days	Vickers Microhardness (VHN)
Rai, P.M./2019/India [32]	Sixty human maxillary premolar teeth	artificial caries formation	CPP-ACPF MI Paste Plus (10) Beta-TCP (ClinPro) (10) Hydroxyapatite (ReminPro) (10)	without treatment and washed with desionized water (10)	14 days	Vickers Microhardness (VHN)
Rana, N./2021/Punjab [33]	Eighty extracted caries-free permanent premolars	Silverstone’s cariogenic solution for four weeks to induce artificial demineralization	Infiltrante de resina (Icon Infiltrant) (10) CPP-ACPF MI Paste Plus (10) NovaMin-fluoride toothpaste (10)	without treatment and washed with desionized water (10)	14 days	Scanning electron microscopy with an energy dispersive X-ray analysis attachment (SEM-EDAX) testing Vickers Microhardness (VHN)
Reise, M./2021/Germany [34]	Two hundred -forty caries-free human teeth (thirdmolars)	artificial demineralization for 14 days	CPP-ACP MI Paste (20) CPP-ACPF MI Paste Plus (20) amine fluoride (ElmexCariesProtection 1400 pp) (20) NaF (Sensodyne Pronamel 1450 ppm) (20)	distilled wáter (20)	7 days	Polarized Light Microscopy (PLM)
Rirattanapong, P./2014/Thailand [35]	Forty-eight sound extracted or naturally exfoliated human primary incisor	caries like lesion formation 60–150 μm deep	Duraphat^®^ Varnish (12) ClinproTM White Varnish (12) TCP-fluoride varnish (12)	deionized water (12)	7 days	Polarized Light Microscopy (PLM)
Rirattanapong, P./2015/Thailand [36]	Thirty-six sound human primary incisors	artificial caries lesion formation 60–100 μm deep	0.05%NaF plus TCP (12) 0.05%NaF (12)	deionized water (12)	7 days	Polarized Light Microscopy (PLM)
Rirattanapong, P./2015/Thailand [37]	Thirty-six human primary incisors	artificial caries lesion formation	0.05% sodium fluoride 20 ppm tricalcium phosphate mouthrinse (12) 0.05% sodium fluoride mouthrinse (12)	deionized water (12)	7 days	Vickers Hardness (VHN)
Rirattanapong, P./2016/Thailand [38]	Fifty anterior teeth	artificial carious lesions approximately 60–100 μm deep	CPP-ACP MI Paste (10) 0.11% Sodium fluoride (500 ppm F) Toothpaste (Colgate^®^ Ultimate Spiderman, Colgate Palmolive Ltd., New York, NY, USA) (10) Nonfluoridated children toothpaste containing DCPD, calcium lactate, and calcium pyrophosphate (Pureen^®^, AmLion Toothpaste) (10) TCP toothpaste (Faculty of Dentistry, Mahidol University, Thailand) (10)	deionized water (10)	7 days	Polarized Light Microscopy (PLM)
Rirattanapong, P./2017/Thailand [39]	Forty human primary incisors	artificial caries lesion formation for 4 days to produce carious lesions 60–100 μm deep	1000 ppm F dentifrice (10) 500 ppm F+TCP dentifrice (10) 1000 ppm F+TCP dentifrice (10)	deionized wáter (10)	7 days	Polarized Light Microscopy (PLM)
Rirattanapong, P./2019/Thailand [40]	Thirty-six human sound primary incisors	for 4 days to produce carious lesions with a depth of 60–100 µm	0.02% sodium fluoride mouthrinse (12) 0.05% fluoride mouthrinse (12)	deionized wáter (12)	7 days	Polarized Light Microscopy (PLM)
Sebastian, R./2022/India [41]	Twenty sound human primary molars	incipient lesions for 2 days	CPP-ACP MI Paste (10) Nano-Hydroxyapatite paste (nano-HAP) (10) Calcium Sucrose Phosphate paste (10)	without treatment and washed with distilled wáter (10)	7 days	Knoop Hardeness (KHN)
Siripipat, J./2017/Thailand [42]	Thirty human primary incisor	white spot lesion formation	5% sodium fluoride with ACP (Enamel Pro^®^) single application (10) 5% sodium fluoride with ACP (Enamel Pro^®^) three applications (10)	distilled wáter (10)	7 days	Vickers Microhardness (VHN)
Tulumbaci, F./2019/Turkey [43]	Seventy primary and Seventy permanent freshly extruded molar	artificial caries	Colgate Cavity Protection (20) Sensodyne Rapid Relief (20) GC MI Paste Plus (20) Clinpro Tooth Creme (20) Clinpro 5000 (20) Sensodyne Repaır and Protect (20)	deionized water (20)	4-week	DIAGNOdent laser fluorescence Polarized Light Microscopy (PLM)
Veeramani, R./2021/India [44]	Sixty Premolars	white spot lesions for a period of 96 h (demineralization cycle) to produce lesions of 0.2 × 10^−1^ mm mmdepth	CaSP (20) CaSPþ0.2% NaF (20)	distilled wáter (20)	21 days	Vickers Microhardness (VHN)
Vyavhare, S./2015/India [45]	Twenty-six freshly extracted human permanent maxillary incisor teeth	early artificial carious lesions, according to Ten Cate and Duijsters	Nano hydroxyapatite 10% (24) CPP-ACP MI Paste (24) Fluoride 1000 ppm (24)	deionized wáter (24)	3, 6, 9 y 12 days	Vickers Microhardness (VHN)
Yu, O.Y./2018/China [46]	Forty-eight human enamel	six days to create subsurface caries lesions	38% SDF solution (Saforide) followed by 5% NaF varnish (Duraphat; Colgate-Palmolive Co.) (12) 38% SDF solution (12) 5% NaF varnish (12)	deionized water (12)	21 days	SEM, EDS, XRD, TEM, XPS

**Table 4:** Characteristics of included agents.

**Agent**	**Company**	**Presentation**	**Active Ingredient**
Duraphat^®^	Duraphat^®^, Colgate-Palmolive GmbH, Waltrop, Germany	varnish	5% NaF Sodium Fluoride (500 ppmF)
MI Varnish™	MI Varnish™, GC Corporation, Hongo, Tokyo, Japan)	varnish	5% NaF with CPP-ACP Sodium Fluoride (500 ppmF) with Casein Phosphopeptide Amorphous Calcium Phosphate
White Vanish™	Vanish^TM^, 3M ESPE, St. Paul, MN, USA	varnish	5% NaF with TCP Sodium Fluoride (500 ppmF) with Tri-calcium Phosphate with Sodium Fluoride
MI Paste	GC, Tokyo, Japan	mousse	CPP-ACP 10% Casein Phosphopeptide Amorphous Calcium Phosphate
MI Paste Plus	GC, Japan	mousse	CPP-ACPF 0.2% (*w*/*w*) (900 ppm) Sodium Fluoride in addition to 10% Casein Phosphopeptide Amorphous Calcium Phosphate
Sensodyne Repair and Protect	Haleon, GlaxoSmithKline, Brentford, UK	toothpaste	Fluoride estanoso Contains NovaMin Technology
Sensodyne Rapid Relief	Haleon, GlaxoSmithKline, UK	toothpaste	Fluoride estanoso
Clinpro	3M ESPE	cream	NaF/fTC 0.21% sodium fluoride with Tri-calcium Phosphate
Clinpro 5000	3M ESPE	cream	NaF/fTC 1.1% sodium fluoride with Tri-calcium Phosphate
ReminPro	Voco, DE	cream	NaF/NHA Fluoride (1450 ppm), Hydroxyapatite and Xilitol
Colgate Sensitive Plus Pro-Argin^TM^	Colgate-Palmolive	toothpaste	8% Arginine bicarbonate, sodium monofluorophosphate, and 1450 ppm calcium carbonate
Colgate^®^ Cavity Protection (Regular)	Colgate-Palmolive	toothpaste	NaF 0.22% sodium fluoride or 1000 ppm F
Colgate^®^ Ultimate Spiderman	Colgate Palmolive	toothpaste	NaF 0.11% Sodium fluoride (500 ppm F)
Regenerate Enamel Science NR-5 Technology	Grupo Unilever	toothpaste	Calcium silicate, sodium phosphate, and sodium monofluorophosphate
BioRepair^®^	Biobel	toothpaste	Zinc hydroxyapatite crystals
2.5% arginine solution	National Basic Research Program of China	solution	2.5% arginine
NaF	National Basic Research Program of China	solution	500 ppm F
Arginine with Sodium Fluoride	National Basic Research Program of China	solution	2.5% arginine/NaF 500 ppmF
Novamin	SHY NM, Group Pharmaceuticals Ltd., Mumbai, India	toothpaste	CSP calcium sodium phosphosilicate
Nano-HAP paste	FGM Brezil	toothpaste	NaF/NHA Hydroxyapatite and 9000 ppm of fluoride
Topex^®^ APF gel	Sultan Healtcare	Fluoride gel	2.0% Sodium Fluoride 0.9% Fluoride Ion
Fluor Opal Varnish White	Ultradent	varnish	xylitol sweetened, 5% sodium fluoride
Enamel Pro	Laboratorios Zeyco S.A. de C.V.	professional prophylactic paste	NaF/ACP 5% sodium fluoride with and amorphous calcium phosphate
Apagard^®^	Sangi, Tokyo, Japan	toothpaste	nano-HA Nano hidroxyapatite
Pureen^®^	AmLion Toothpaste Mfg., Petaling Jaya, Malaysia	nonfluoridated children toothpast	8% Xylitol, calcium lactate, and calcium pyrophosphate
Dokbuaku^®^	Kids, Twinlotus Co., Ltd., Bangkok, Thailand	non-fluoridated toothpaste	CaGP and CL Calcium glycerophosphate (CaGP), an organic polyphosphate
Apa Cared	Flüssiger Zahnschmelz	toothpaste	NHAPF 1% nano-HAP + 1450 ppm F/ NaF
Blend-a Mede	Blend-a Mede	toothpaste	1450 ppm F/NaF
F5000 ControlRx	3M ESPE	toothpaste	NaF 1.1% NaF dentifrice, 5000 ppm
Enafix	Global Calcium	toothpaste	CaSP Calcium Sucrose Phosphate
Curasept ADS 205	Curaden International AG, Kriens, Switzerland	mouthwash	NaF 0.05% NaF and chlorhexidine 0.05%

## Data Availability

The data presented in this study are available upon reasonable request from the corresponding author.
